# A CRISPR/Cas9-induced male-sterile line facilitating easy hybrid production in polyploid rapeseed (*Brassica napus*)

**DOI:** 10.1093/hr/uhae139

**Published:** 2024-05-28

**Authors:** Mengxin Tu, Ruisen Wang, Wenhui Guo, Shiqi Xu, Yang Zhu, Jie Dong, Xiangtan Yao, Lixi Jiang

**Affiliations:** Institute of Crop Science, Zhejiang University, Hangzhou 310058, China; Institute of Economic Crops, Jiaxing Academy of Agricultural Sciences, Jiaxing 314016, China; Institute of Crop Science, Zhejiang University, Hangzhou 310058, China; Institute of Crop Science, Zhejiang University, Hangzhou 310058, China; Institute of Crop Science, Zhejiang University, Hangzhou 310058, China; Institute of Crop Science, Zhejiang University, Hangzhou 310058, China; Institute of Economic Crops, Jiaxing Academy of Agricultural Sciences, Jiaxing 314016, China; Institute of Crop Science, Zhejiang University, Hangzhou 310058, China

## Abstract

Rapeseed is a globally significant oilseed crop cultivated to meet the increasing demand for vegetable oil. In order to enhance yield and sustainability, breeders have adopted the development of rapeseed hybrids as a common strategy. However, current hybrid production systems in rapeseed have various limitations, necessitating the development of a simpler and more efficient approach. In this study, we propose a novel method involving the targeted disruption of *Defective in Anther Dehiscence1* of *Brassica napus* (*BnDAD1*), an essential gene in the jasmonic acid biosynthesis pathway, using CRISPR/Cas9 technology, to create male-sterile lines. *BnDAD1* was found to be dominantly expressed in the stamen of rapeseed flower buds. Disrupting *BnDAD1* led to decreased levels of α-linolenic acid and jasmonate in the double mutants, resulting in defects in anther dehiscence and pollen maturation. By crossing the double mutant male-sterile lines with male-fertile lines, a two-line system was demonstrated, enabling the production of *F*_1_ seeds. The male-sterile trait of the *bndad1* double mutant lines was maintainable by applying exogenous methyl jasmonate and subsequently self-pollinating the flowers. This breakthrough holds promising potential for harnessing heterosis in rapeseed and offers a simpler and more efficient method for producing hybrid seeds.

## Introduction

Hybrid vigor, also known as heterosis, describes the phenomenon where the offspring resulting from a cross between two inbred lines surpasses the performance of the parent lines [1]. Heterosis has proven to be particularly impactful in inbred species, as it can substantially increase yield by 20% to over 50% [2]. Rapeseed (*Brassica napus*) is a globally significant oilseed crop cultivated to meet the escalating demand for vegetable oil. The adoption of hybrids has played a key role in significantly enhancing the yield and overall production of rapeseed, especially in major production regions like Canada, China, and Europe [3, 4]. In rapeseed, the primary approach for hybrid production relies on the utilization of male sterility (m-s), which is crucial for harnessing the benefits of heterosis [5].

Male sterility refers to the abnormal development of stamens during sexual reproduction in higher plants [6]. Its existence was initially observed in tobacco species and interspecific crosses, and it was subsequently utilized in various plant species, including *Oryza sativa*, *Zea mays*, *B. napus*, *Gossypium hirsutum*, and *Glycine max* [1, 7]. Male sterility is categorized into cytoplasmic male sterility (CMS) and genic male sterility (GMS) [8]. CMS is typically controlled by mitochondrial genes and a few nuclear genes, and it is commonly used for hybrid seed production with a three-line system [9]. CMS eliminates the need for anther removal and simplifies the identification of maintainers of m-s [5, 10]. Consequently, numerous CMS systems in rapeseed (*B. napus*) were developed, including *Ogura* CMS (*Ogu* CMS), *Polima* CMS (*Pol* CMS), *Nap* CMS, *Kosena* CMS (*Kos* CMS), *Tour* CMS, and others [11, 12]. Among them, the *Pol* and *Ogu* systems are the most extensively used CMS systems in *B. napus*. However, CMS systems face intrinsic challenges. For example, the *Pol* system displays unstable m-s due to environmental factors [13], while the *Ogu* system encounters difficulties in finding suitable restorers [14]. Additionally, limited genetic diversity and increased disease susceptibility pose limitations to the development and application of CMS systems [8].

GMS is primarily controlled by one or more nuclear genes, and its utilization in hybrid breeding typically involves a two-line system for seed production [1, 15]. While GMS traits cannot be efficiently maintained compared with CMS, the discovery of environment-sensitive GMS (EGMS) mutants has enabled the utilization of certain GMS characteristics in hybrid breeding programs [16]. EGMS includes photoperiod-sensitive GMS (PGMS), reverse PGMS, and temperature-sensitive GMS (TGMS), wherein the mutant line of EGMS is propagated through self-pollination when grown under specific permissive conditions [1]. GMS offers distinct advantages, such as stable and complete m-s, and ease of transferability of m-s genes. However, there are also evident drawbacks. During seed production, the offspring of GMS plants pollinated by heterozygous pollinators always segregate in a 1:1 ratio, necessitating the manual removal of fertile plants [5].

There is an increasing demand for the development of a simpler and more efficient system to promote hybrid production in rapeseed. Jasmonic acid (JA) is a naturally occurring growth regulator found in higher plants [17]. JAs play crucial roles in various physiological processes, including defense against pathogen infection, tolerance to abiotic stress, and multiple developmental processes such as anther dehiscence, leaf senescence, and root growth [18]. Enzymes involved in the JA biosynthesis pathway have been identified. JAs are synthesized from α-linolenic acid (α-LeA) through the octadecanoid pathway [18, 19]. α-LeA is released from galactolipids in the chloroplast thylakoid membrane by phospholipase A1 (PLA) and transported into plastids [20]. Controlled by 13-lipoxygenase (LOX), allene oxide synthase (AOS), and allene oxide cyclase (AOC), α-LeA is converted to (9*S*,13*S*)-12-oxo-phytodienoic acid (OPDA). OPDA is then transported to peroxisomes, where it undergoes reduction, activation, and β-oxidation to produce JA [19]. Subsequently, JA is transported to the cytoplasm, where it can form bioactive (+)-7-iso-JA-Ile or undergo metabolism to inactive forms through methylation, glucosylation, or sulfation [21]. Loss-of-function mutations in multiple genes involved in the JA biosynthesis pathway resulted in various m-s mutants [22, 23]. Specifically, *Defective in Anther Dehiscence1* (*DAD1*) encodes a phospholipase A1 lipolytic enzyme that is crucial for the release of α-LeA and the provision of α-LeA for JA biosynthesis [22]. The *Arabidopsis dad1* mutant exhibits defects in anther dehiscence, pollen maturation, and flower opening, resulting in absolute m-s. Notably, this type of m-s can be restored by the application of exogenous methyl jasmonate (MeJA) [22]. While the discovery of the *dad1* phenotype in *Arabidopsis* has valuable implications for creating m-s lines in rapeseed, it is essential to acknowledge that rapeseed is an allotetraploid with a more complex flowering mechanism and a longer flowering duration compared with *Arabidopsis* [6, 24]. Therefore, the challenge remains in knocking out *BnDAD1* and assessing the expected outcomes in rapeseed.

In this study, we propose a novel approach to create m-s lines by specifically disrupting *BnDAD1*, an essential gene in the JA biosynthesis pathway, using CRISPR/Cas9 technology. By placing these m-s lines in close proximity to male-fertile lines, a two-line system was demonstrated, facilitating the production of *F*_1_ seeds. The m-s was maintained by applying exogenous MeJA and subsequently self-pollinating the flowers. This innovative approach offers an efficient strategy for promoting hybrid production in rapeseed and holds significant potential for commercial applications.

## Results

### Expression pattern of *DAD1* in rapeseed

**Figure 1:**
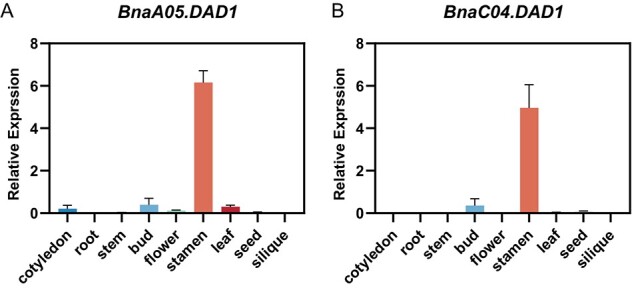
Expression pattern of *BnDAD1* in rapeseed. Relative gene expression of *BnaA05.DAD1* (**A**) and *BnaC04.DAD1* (**B**) in diverse rapeseed tissues was examined by RT–qPCR. Relative transcript levels were determined using the 2^–ΔΔCt^ method, with *BnaACTIN7* serving as the internal control. Values are means ± standard deviations of three biological replicates.

In the spring-ecotype variety of *B. napus* known as ‘Westar’, two orthologous copies of the *Arabidopsis DAD1* gene, specifically *BnaA05.DAD1* (*BnaA05G0038100WE*) and *BnaC04.DAD1* (*BnaC04G0053500WE*), each comprising a single exon, were identified through BLAST analysis using the amino acid sequence of *AtDAD1* (Supplementary Data Fig. S1A). Concurrently, the two copies of *BnDAD1* were also identified in other rapeseed varieties such as ‘Tapidor’, ‘ZS11’, and ‘Shengli’, with these homologs displaying a significant degree of sequence conservation in their coding regions, implying a functional similarity in the encoded enzymes (Supplementary Data Fig. S1B). To elucidate the expression profile of *BnDAD1* in rapeseed, RT–qPCR was conducted on RNA isolated from various tissues of ‘Westar’. Interestingly, the expression pattern of *BnDAD1* closely resembled that of *DAD1* in *Arabidopsis*, as evidenced by the striking similarities observed (Fig. 1) [22]. Particularly noteworthy was the observation of high transcription levels of the genes in the stamens of flower buds in both species.

### Overexpression of *BnDAD1* in *Arabidopsis* caused defects in chloroplasts

DAD1 was identified as a phospholipase A1 enzyme, known for its ability to hydrolyze phosphatidylcholine, as well as monogalactosyldiacylglycerol and trilinolein at a lower rate [25]. It belongs to class I PLA1 and is localized in chloroplasts [22]. *DAD1* exhibits a widespread presence across various species, such as *Arabidopsis thaliana*, rapeseed (*B. napus*), maize (*Z. mays*), and rice (*O. sativa*) (Supplementary Data Fig. S2). In terms of genetic similarities, the *BnaA05.DAD1* in rapeseed can be traced back to *Brassica rapa*, while the *BnaC04.DAD1* originates from *Brassica oleracea* (Supplementary Data Fig. S2A) [24]. Notably, when compared with orthologs in other plant species, *BnDAD1* demonstrates the highest degree of genetic similarity to that of *Arabidopsis*. To investigate whether *BnDAD1* retains its conserved function with *AtDAD1*, we overexpressed the coding sequence of both *BnaA05.DAD1* and *BnaC04.DAD1*, as well as *AtDAD1*, in *Arabidopsis* (Col-0) under the control of the cauliflower mosaic virus 35S promoter. *T*_1_ seedlings of all three overexpressing lines exhibited a range of pale color phenotypes. Some seedlings displayed a complete loss of green pigment, leading to their demise before true leaf formation compared with the wild type (WT) (Supplementary Data Fig. S3A–D), while some other seedlings displayed white patches in their leaves (Supplementary Data Fig. S3E). These findings strongly indicate that both *BnaA05.DAD1* and *BnaC04.DAD1* exhibit conserved functions similar to *AtDAD1*, thereby influencing the functionality of chloroplasts.

### Targeted mutagenesis of *BnDAD1*

**Figure 2:**
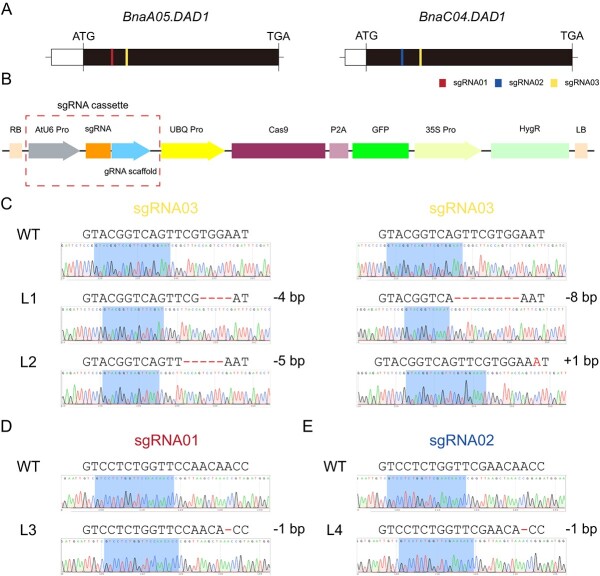
CRISPR/Cas9 induced mutants of *BnDAD1* in rapeseed. **A** Gene models of *BnaA05.DAD1* and *BnaC04.DAD1*, each of which comprises one exon (white box, 5′-UTR region; black box, coding sequence region). sgRNA01, sgRNA02, and sgRNA03 are represented by red, blue, and yellow lines, respectively. **B** CRISPR/Cas9 construct containing an sgRNA cassette with the *Arabidopsis* U6-26 promoter, sgRNA, and gRNA scaffold; a Cas9-P2A-GFP expression cassette controlled by the *Arabidopsis Ubiquitin-10* promoter; and a hygromycin resistance cassette driven by the cauliflower mosaic virus 35S promoter. **C**–**E** Sequences of sgRNAs and sequencing results of target sites in L1 and L2 DM lines (**C**), a *bna.a05.dad1* SM (L3) line (**D**), and a *bna.c04.dad1* SM (L4) line (**E**) compared with the WT. InDels are marked in red, with details provided on the right.

To generate mutants with silenced *DAD1* copies in rapeseed, we designed three single-guide RNAs (sgRNAs). sgRNA01 and sgRNA02 were designed to target *BnaA05.DAD1* and *BnaC04.DAD1*, respectively, while sgRNA03 targeted conserved sequences in both homologs (Fig. 2A). These sgRNAs were strategically positioned in the exon region near the start codon, which is expected to induce frameshift mutations throughout the entire gene sequence. We constructed three CRISPR/Cas9 vectors based on a genome-editing vector described in a previous report [26]. Each construct contained a specific sgRNA driven by the *AtU6-26* promoter, while the Cas9-P2A-GFP cassette was driven by the *UBQ10* promoter (Fig. 2B). These constructs were separately transferred into ‘Westar’ via the *Agrobacterium*-mediated transformation method, resulting in the successful generation of 17, 13, and 20 transgenic lines positive for sgRNA01, sgRNA02, and sgRNA03, respectively, in the *T*_0_ generation.

To obtain stable lines with targeted mutations, we subjected the plants to screening using a 25 mg/l hygromycin B solution. DNA was then extracted from positive *T*_1_ and *T*_2_ seedlings, and PCR was performed to amplify the genomic regions flanking the target sites. Subsequently, Sanger sequencing was conducted to determine the nucleotide sequences. Through this process, we identified four homozygous *bndad1* mutants, including two double mutants (DMs) (L1 and L2), one *BnaA05.dad1* single mutant (SM) (L3), and one *BnaC04.dad1* SM (L4) (Fig. 2C–E). All of these mutants exhibited frameshift mutations, resulting in the production of non-functional DAD1 proteins (Supplementary Data Fig. S4).

### Decrease of the levels of α-linolenic acid and jasmonates in double mutants

**Figure 3:**
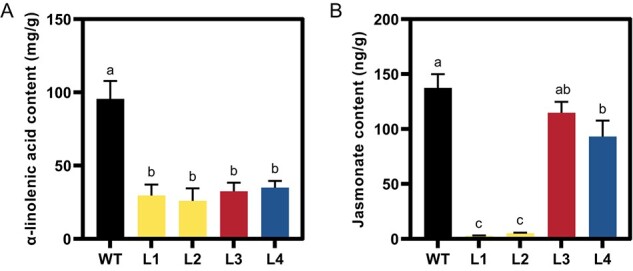
Comparison of content of α-LeA and JA in WT and four mutants. **A** α-LeA content in flower buds of WT, L1, L2, L3, and L4 measured by GC. **B** JA content in flower buds of WT, L1, L2, L3, and L4 determined by HPLC–MS/MS. Data represent the sum of both compounds. Values in the bars represent means of three biological replicates, and error bars indicate the standard deviations. Different lowercase letters above the bars indicate statistical significance at *P* < 0.05 based on Student’s *t*-test.

DAD1 plays a pivotal role in the biosynthesis pathway that converts trienoic fatty acids into α-LeA, which is subsequently translocated into plastids and contributes to the production of JA via the octadecanoid pathway [18, 21]. Loss-of-function mutations in *DAD1* were anticipated to disrupt the synthesis of downstream metabolites. Consequently, we analyzed the α-LeA content in flower bud clusters using gas chromatography (GC). The findings unveiled a notable reduction in α-LeA levels across all mutant samples compared with the WT (Fig. 3A).

Furthermore, we performed extraction and quantification of JA and MeJA utilizing high-performance liquid chromatography–tandem mass spectrometry (HPLC–MS/MS). In the DMs (L1 and L2), the concentrations of jasmonates in flower buds were significantly lower compared with the WT (Fig. 3B). The difference in JA levels between the WT and the SMs (L3 and L4) was smaller than the gap between the DMs (L1 and L2) and the WT. In the DMs, the decrease in JA content was stronger than the drop in α-LeA content, while in the SMs the decrease in JA content was relatively weaker than the reduction in α-LeA.

### Defect in anther dehiscence and pollen maturation in the double mutants

**Figure 4:**
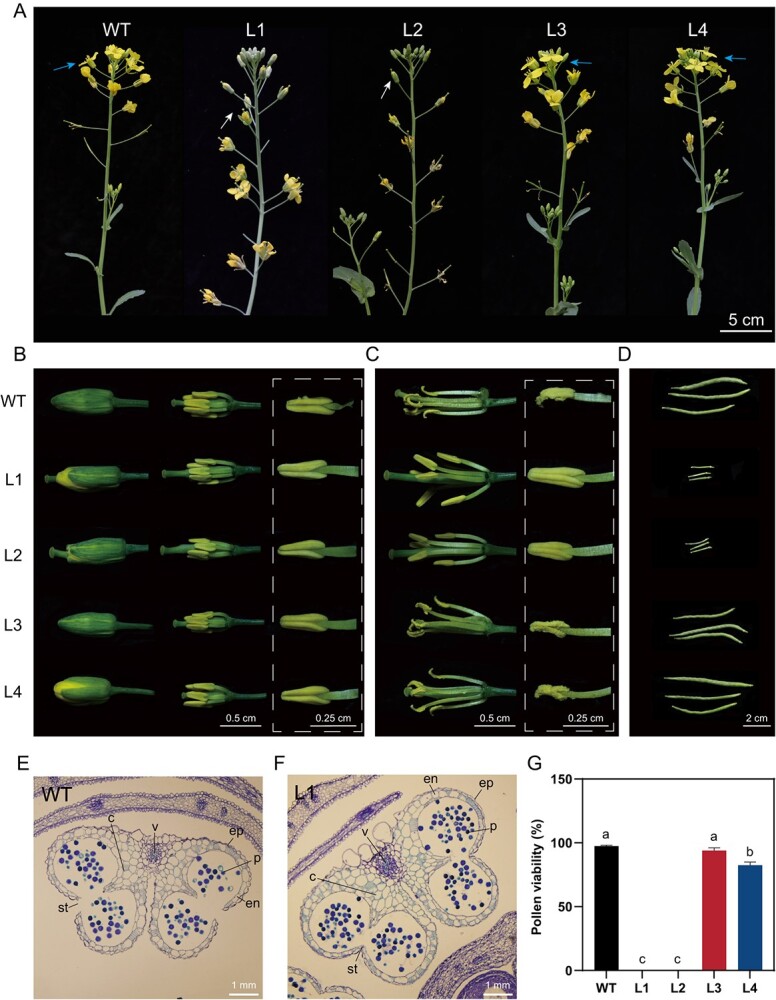
Phenotypic characterization of flower buds, open flowers, and silique development in WT and four mutants. **A** The growth and flowering of the WT and four mutants. Blue arrows indicate normally developed flower buds and opening flowers, while white arrows highlight abnormally developed flower buds and flowers. Scale bar = 5 cm. **B** Flower buds, buds without sepals and petals, and anthers in flower buds before flowering. Scale bar = 0.5 cm; dashed box scale bar = 0.25 cm. **C** Open flowers without sepals and petals, and anthers in fully opened flowers. Scale bar = 0.5 cm; dashed box scale bar = 0.25 cm. **D** Siliques at 12 DAP of WT and the four mutants. Scale bar = 2 cm. **E**, **F** Paraffin sections of transverse anther in flower buds 1–2 days before flowering in WT (**E**) and DM (L1) (**F**). Epidermis (ep), endothecium (en), stomium (st), connective (c), vascular bundle (v), and pollen grains (p). Scale bar = 1 mm. **G** Pollen viability of WT, L1, L2, L3, and L4 assessed after 5 h of room temperature staining with Alexander’s staining solution. Values in the bars represent means of three biological replicates, and error bars indicate standard deviations. Different lowercase letters above the bars indicate statistical significance at *P* < 0.05 based on Student’s *t*-test.

We further examined the phenotypic effects of the mutants. At the seedling stage, all mutants showed normal growth of roots, leaves and stems, compared with the WT. However, after flowering, L1 and L2 plants failed to produce fertile siliques, and exhibited an abnormal flower appearance. Conversely, L3 and L4 displayed growth patterns similar to the WT without any noticeable apparent abnormalities (Fig. 4A). To better observe this abnormal phenotype, we dissected flower buds 1–2 days before flowering. It was evident that in the DMs (L1 and L2) the stigmas extended beyond the position enveloped by the sepals of the flower buds, whereas this was not observed in the WT and the SMs (L3 and L4) (Fig. 4B). Moreover, during the flowering period, the opening flowers of the DMs displayed significantly elongated stigmas compared with the WT. Importantly, the DM anthers were unable to dehisce and release pollen grains, resulting in m-s (Fig. 4C). Moreover, it is noteworthy that the siliques of L1 and L2 were noticeably unfertilized, whereas those of WT, as well as L3 and L4, exhibited evidently normal fertilization and developmental patterns ~12 days after flowering (Fig. 4D).

To investigate whether the dehiscence defect was associated with any morphological abnormalities in the anther tissues, we performed paraffin sections and examined transverse sections of anthers from flower buds of both the WT and a DM (L1). The paraffin sections of the flower buds revealed no structural differences between the DM and the WT anthers before anther dehiscence, except for stomium breakage, which is critical for pollen release (Fig. 4E and F). We stained the pollen with Alexander’s solution to assess pollen viability. After 5 h of room temperature staining, it was evident that the pollen viability of the SMs was comparable to that of the WT, while pollen vitality showed a significant decrease in the DMs compared with the WT (Fig. 4G). Moreover, we conducted reciprocal pollinations between the DMs and the WT. Intriguingly, when the DM plants were used as the maternal parent and the WT plants as the paternal parent, all DM plants exhibited fertility and developed typical siliques. Conversely, when the roles were reversed, with the WT plants as the maternal parent and the DM plants as the paternal parent, abnormal silique development was observed in the WT plants (Supplementary Data Fig. S5). Additionally, we introduced three alternative paternal parents, specifically ‘Golden’, ‘Brutor’, and ‘ZS11’, to pollinate the DM stigmas (maternal parent). In all three hybrid combinations, normal and fertilized siliques were successfully produced (Supplementary Data Fig. S6).

These findings indicate that the m-s observed in the DMs is attributable to the anther dehiscence defect, leading to the failure of pollen grain release. Prior to dehiscence, no structural variances were detected in the anthers of the DMs compared with the WT, suggesting that the developmental processes in the DMs progressed normally until dehiscence, but were impeded at the stage of stomium breakage.

### Recovery of double mutants by application of exogenous methyl jasmonate

**Figure 5:**
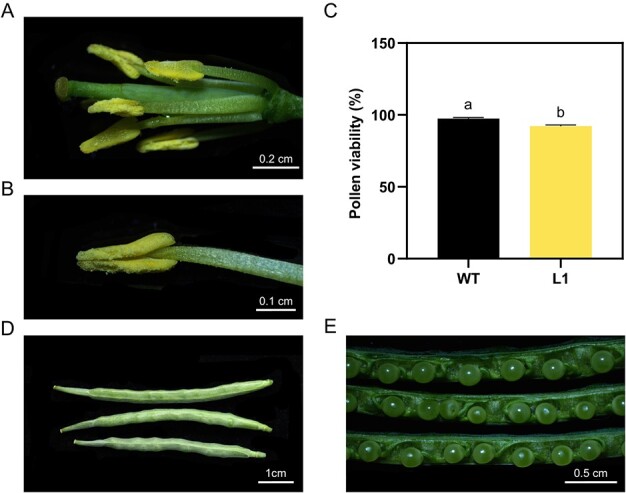
Phenotypic characterization of floral organs, developing siliques, and seeds in L1 after application of exogenous 500 μM MeJA. **A**, **B** Open flowers without sepals and petals (**A**) and anthers (**B**) in L1 after application of 500 μM MeJA. **C** Comparison of pollen viability between WT and L1 treated with 500 μM MeJA solution. Pollen viability was assessed by staining with Alexander’s solution at room temperature for 5 h. Values in the bars represent means of three biological replicates, and error bars indicate standard deviations. Different lowercase letters above the bars indicate statistical significance at *P* < 0.05 based on Student’s *t*-test. **D** Siliques at 12 DAP after application of 500 μM MeJA. Scale bar = 1 cm. **E** Seeds in siliques at 12 DAP after application of 500 μM MeJA. Scale bar = 0.5 cm.

Previous studies demonstrated that *Arabidopsis* mutants deficient in JA biosynthesis, including *fatty acid desaturase 3* (*fad3*) *fad7 fad8*, *lipoxygenase 3* (*lox3*), *lox4*, *oxophytodienoate-reductase 3* (*opr3*), and *dad1*, display m-s characterized by arrested stamen development at anthesis. However, the fertility of these mutants could be restored by the exogenous application of JA [22, 23, 27, 28]. Considering the conserved functions of *BnDAD1* and *AtDAD1*, we investigated whether the m-s of DM (L1) could be rescued by the application of MeJA. Indeed, after immersing unopened flower bud clusters in a 500-μM MeJA solution for 0.5 min daily or spraying unopened flower bud clusters with a 500-μM MeJA solution daily for 1 week, we observed anther dehiscence and pollen release in the DM (L1) plants (Fig. 5A and B). Furthermore, we evaluated pollen viability, which was found to be similar to that of the WT following treatment with JA (Fig. 5C). Of greater significance, siliques containing fully developed seeds, indicative of successful pollination, demonstrated full restoration of anther fertility in the DM plants at 12 days after pollination (DAP) (Fig. 5D and E). More importantly, the progeny of restored L1 with the application of MeJA all exhibited sterility.

## Discussion

### Defect of jasmonic acid biosynthesis that causes male sterility in rapeseed

In this study, we used CRISPR/Cas9 genome editing technology to knock out *BnDAD1* and create m-s lines. We grew the *bndad1* m-s lines near male-fertile lines to produce *F*_1_ hybrid seeds. To maintain the m-s feature of *bndad1* mutants, we treated the unopened flowers with MeJA and then self-pollinated them. We named this rapeseed hybrid utilization system the *bndad1* two-line system. *DAD1* is an important gene involved in JA biosynthesis and is conserved across different plant species, including *A. thaliana*, rapeseed, rice, maize, and soybean, among others (Supplementary Data Fig. S2). *DAD1* orthologs in these species share significant sequence homology and likely have similar functions in regulating anther dehiscence, specifically by affecting JA biosynthesis.

Anther dehiscence is a complex process that involves the activation of specific genes and the synthesis and accumulation of enzymes and other proteins [29, 30]. These proteins weaken and break down the cell walls of the stomium, a specialized tissue in the anther that acts as a barrier to prevent the premature release of pollen [31]. JA signaling activates downstream genes that promote the synthesis of enzymes responsible for degrading the stomium cell walls, leading to stomium rupture and pollen release. In the case of the DM *bndad1*, a defect in JA synthesis disrupts this regulation, failing to synthesize and accumulate the necessary enzymes. As a result, the stomium remains intact and prevents the release of pollen. The substantial impact of the *bndad1* double mutation on JA content rather than α-LeA content comes as a somewhat unexpected finding. One potential explanation for this observation could be that when the production of JA, the final product of a cascade of biochemical reactions, has a sharp decrease, it might feedback-trigger a compensatory increase in the generation of intermediate precursors, such as α-LeA, in response to this perturbation.

It is worth noting that anther dehiscence is influenced by multiple factors, and JA is just one component. Other hormone signaling pathways, such as ethylene and gibberellins, also play significant roles in anther dehiscence and could potentially compensate for the defect in JA [32–34]. In our research with *B. napus*, this kind of substitution from other hormones in regulating anther dehiscence was not observed. This does not imply that the loss of function in *DAD1* orthologs, which may result in the inhibition of JA synthesis, would necessarily cause anther dehiscence failure in other crop species. Each species should be investigated independently. It is possible that in some species, if JA synthesis is blocked, ethylene or gibberellins may become the dominant factors regulating anther dehiscence, thus not causing a failure in pollen release. It is worth noting that JA is known to exert diverse roles in plant development. However, as depicted in Fig. 1, the expression of *BnDAD1* exhibits significantly higher levels in the buds compared with other tissues. Therefore, it is plausible to suggest that the disruption of *BnDAD1* has a lesser impact on JA synthesis and physiological consequences in other tissues, potentially due to distinct mechanisms governing JA synthesis in those tissues.

In our study, we found that overexpression of either *BnaA05.DAD1* or *BnaC04.DAD1* driven by the 35S promoter of the cauliflower mosaic virus in *Arabidopsis* resulted in defects in chloroplast synthesis, as evidenced by the presence of albino leaves and seedlings (Supplementary Data Fig. S3). This suggests underlying mechanisms at play. Firstly, *BnDAD1* overexpression may interfere with the biogenesis of chloroplasts, which involves processes such as protein import, assembly of photosynthetic complexes, and pigment synthesis. This interference can lead to reduced chlorophyll levels and result in albino leaves and seedlings [35–38]. Secondly, chloroplast dysfunction can lead to the production of excessive reactive oxygen species (ROS), causing cellular damage. Therefore, *BnDAD1* overexpression may disrupt the normal redox balance within chloroplasts, leading to increased ROS accumulation and subsequent damage to chloroplast components [39, 40]. What is more, ROS participates in the signal transduction networks during programmed cell death [41, 42]. Thus, we make a deduction that *BnDAD1* overexpression may cause defects of the membrane and disrupt protein homeostasis within chloroplasts or disturb the balance of cell death regulators, impairing the functioning of essential chloroplast proteins and resulting in albino phenotypes. Further investigation, such as transcriptomic analysis, protein profiling, and characterization of related mutants, is required to confirm these mechanisms and explore the precise molecular pathways affected by *BnDAD1* overexpression in chloroplasts.

### Knocking out multiple copies of *DAD1* in the tetraploid rapeseed genome

In our study, we identified two homologs of *DAD1* in rapeseed, namely *BnaA05.DAD1* and *BnaC04.DAD1* (Supplementary Data Fig. S1). Anther indehiscence was only observed in the DM plants, where both *BnaA05.DAD1* and *BnaC04.DAD1* lost their function. Knocking out only one of them did not result in anther indehiscence or m-s (Figs 2 and 4). *Brassica napus* is a polyploid crop that originated from the hybridization of two diploid progenitors, *B. rapa* (AA) (*n* = 10) and *B. oleracea* (CC) (*n* = 9) <7500 years ago [24]. An *Arabidopsis* gene typically has two to nine orthologs in its polyploid relative *B. napus*, located in either the A subgenome or the C subgenome [43]. Research on biased expression between subgenomes in polyploid crops has provided insights into gene regulation and its impact on crop traits [44–46]. Transcriptomic approaches, such as RNA sequencing (RNA-seq), were used to compare gene expression levels in different tissues or under various conditions. These studies showed that a significant proportion of genes exhibit biased expression, with some being preferentially expressed in one subgenome over the other. To understand the mechanisms underlying biased expression, various factors were proposed, including dosage sensitivity and epigenetic regulation involving DNA methylation and histone modifications [47].

Designing sgRNAs to target and knock out multiple homologous copies of a gene in a polyploid species can be challenging but feasible. In our study, first we identified all the homologous copies of *BnDAD1* in the genome and then aligned the sequences of *BnDAD1* copies to identify regions of high sequence conservation. We also performed off-target analysis to ensure that the designed sgRNAs were specific to the target copies and did not show significant sequence similarity to other genomic regions. In the case of *BnDAD1*, both its homologs in the A and C subgenomes were highly expressed in stamens of flowers, suggesting that they were both functional (Fig. 1). Knocking out one of them did not achieve the aim of inducing anther indehiscence.

### Advantages of the *bndad1* two-line system over other systems for hybrid production

**Figure 6:**
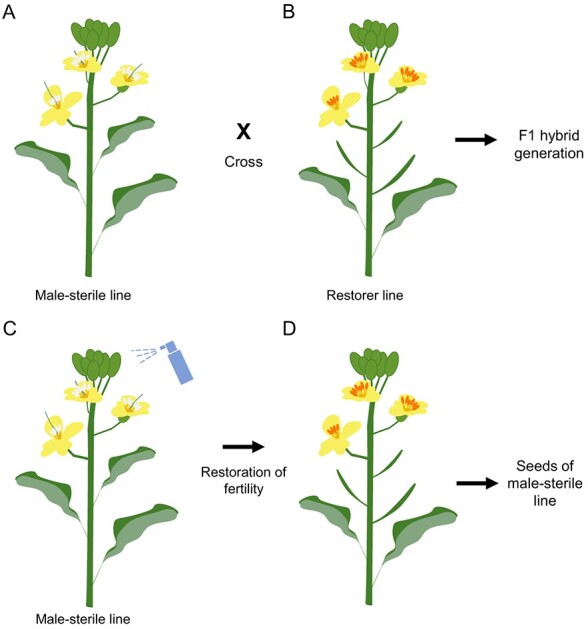
Illustration of a two-line system for hybrid seed production in rapeseed. **A** The m-s line (DM *bndad1*) grown in close proximity to a male-fertile line. **B** A male-fertile line used as a pollen donor to produce *F*_1_ hybrid seeds. **C** An m-s line treated with exogenous MeJA. **D** Recovered male-fertile DM *bndad1* exhibiting normal development of siliques.

In our study, we successfully generated m-s lines (L1 and L2) in rapeseed by disrupting the *BnDAD1* gene. The DM *bndad1* plants showed a failure to release pollen grains from mature anthers (Fig. 4) and had a significantly reduced number of viable pollen grains compared with the WT (Fig. 4G). The fertility of the DM *bndad1* plants was restored when treated with a 500-μM MeJA solution before anthesis (Fig. 5), and the progeny of restored plants after application of MeJA all exhibited sterility. Based on the *bndad1* DM mutant and the successful crossings between the DM and three rapeseed cultivars (Supplementary Data Fig. S6), we propose a two-line system for rapeseed hybrid production (Fig. 6). The advantages of this *bndad1* m-s line over other m-s lines include its stable and complete m-s, independent of environmental conditions such as light and temperature. Moreover, finding a restorer for the *bndad1* line is relatively easy, as almost all fertile rapeseed genotypes can serve as restorers (Supplementary Data Fig. S6). In contrast, identifying a suitable restorer for some CMS-genic lines, such as the *Ogu* m-s lines, requires extensive effort [14]. Additionally, the use of MeJA on rapeseed poses no environmental pollution risks, as neither MeJA nor its precursor, linolenic acid, is toxic to humans. However, the spraying of MeJA solution on flowering buds does require additional manpower. Nowadays, the use of drones can significantly reduce labor expenditure. On the other hand, optimizing the planting ratio of m-s and fertility-restoration lines would further minimize the production cost of *F*_1_ hybrid seeds.

It is noteworthy that the two-line system utilized for rapeseed hybrid production is not an original concept. An alternative type of two-line system in rapeseed is based on self-incompatible (SI) lines. In the SI system, a plant’s pollen grains are incapable of fertilizing its own or closely related plants’ stigmas due to the mutual recognition between stigma and pollen proteins, which results in the prevention of self-pollination. *F*_1_ hybrid seeds can be obtained by crossing an SI line with a complementary fertility-restoration line [48]. However, maintaining the SI two-line system involves substantial labor costs, as manual intervention is required to open the follower petals of unopened buds in the SI line to overcome self-incompatibility and retain the SI trait in subsequent generations. In contrast to the *bndad1* two-line system we have devised, the SI two-line system entails considerably higher production expenses.

## Conclusion

In conclusion, we propose a new approach using CRISPR/Cas9 technology to disrupt the *BnDAD1* gene, which is crucial in the JA biosynthesis pathway. This disruption results in the creation of m-s lines in rapeseed. Our findings indicate that *BnDAD1* is primarily expressed in the stamen of rapeseed flower buds. Disrupting *BnDAD1* leads to reduced levels of α-LeA and JA in the DMs, causing defects in anther dehiscence and pollen-grain release. By placing the m-s lines alongside male-fertile lines, we demonstrated a two-line system that allows the production of *F*_1_ seeds. The m-s trait of the *bndad1* DM lines can be maintained by applying exogenous MeJA and subsequently self-pollinating the flowers. This breakthrough has the potential to enhance heterosis in rapeseed and offers a simpler and more efficient method for producing hybrid seeds.

## Materials and methods

### Plant materials

In this study we utilized ‘Westar’ (AACC, 2*n* = 38), a spring ecotype of *B. napus* from Canada, as the plant material for transformation and experiments. All plants were cultivated in a greenhouse located at Zijingang Campus, Zhejiang University, Hangzhou, China.

### RNA isolation and real-time quantitative PCR

Total RNAs of cotyledon, root, stem, bud, flower, stamen, leaf, seed, and silique were extracted from *B. napus* cv. ‘Westar’ using the FastPure Universal Plant Total RNA Isolation Kit (Vazyme Biotech Co., Ltd). Subsequently, cDNA synthesis was performed using the HiScript II Q RT SuperMix for qPCR (+gDNA wiper) (Vazyme Biotech Co., Ltd). Quantitative PCR (qPCR) was conducted using the Taq Pro Universal SYBR qPCR Master Mix (Vazyme Biotech Co., Ltd). To ensure robustness and reliability, three independent biological replicates, each with three technical replicates, were included. The relative transcript levels were determined using the 2^–ΔΔCt^ method, with *BnaACTIN7* serving as the internal control. The primer sequences used for the real-time quantitative PCR (RT–qPCR) are provided in Supplementary Data Table S1.

### Overexpression of *AtDAD1* and *BnDAD1* in *Arabidopsis*

The coding sequences of *BnaA05.DAD1* and *BnaC04.DAD1* were obtained from ‘Westar’, while the coding sequence of *AtDAD1* was isolated from Col-0. These sequences were subsequently inserted into the pCambia1300-35S-GFP vector under the control of the 35S promoter. The resulting three constructs, containing 35S::*AtDAD1*, 35S::*BnaA05.DAD1*, and 35S::*BnaC04.DAD1*, were then respectively introduced into *Agrobacterium tumefaciens* strain GV3101 and subsequently transformed into the Col-0 plant using the floral dip method [49]. To select for successful transformation, *T*_1_ seeds were screened on MS medium supplemented with 50 mg/l hygromycin B. Primers used are listed in Supplementary Data Table S1.

### Design of sgRNAs and construction of CRISPR/Cas9 vectors

Target-specific sgRNAs were designed using CRISPR-P (http://crispr.hzau.edu.cn/CRISPR/). For this experiment, three sgRNAs were selected, and their sequences can be found in Supplementary Data Table S1. To ensure accuracy, all three sgRNAs were assessed in ‘Westar’ by performing PCR and Sanger sequencing using specific primers. This step was crucial to confirm the absence of any polymorphisms in the selected sgRNAs and their corresponding target sites.

To generate the CRISPR/Cas9 plasmids, we followed the protocol previously described in a previous report [26]. Three separate plasmids were constructed, each containing one of the three sgRNAs. In each plasmid, a sgRNA cassette was composed of the *Arabidopsis* U6-26 promoter, the sgRNA sequence, and the gRNA scaffold. Additionally, a Cas9-P2A-GFP expression cassette, driven by the *Arabidopsis Ubiquitin-10* promoter, was included in every construct. A hygromycin resistance cassette, driven by the cauliflower mosaic virus 35S promoter, was also incorporated. The oligos and primers used for the construction of the CRISPR/Cas9 vectors can be found in Supplementary Data Table S1.

### *Agrobacterium*-mediated transformation of rapeseed

Three CRISPR/Cas9 vectors were separately transformed into ‘Westar’ using the *A. tumefaciens*-mediated hypocotyl method described in a previous study [50], with certain modifications. The regenerated transformants containing roots (*T*_0_) were subsequently transferred to soil and then transferred to a greenhouse for further growth and development.

### Identification of mutant transgenic plants

Genomic DNA from transgenic seedlings was isolated using the standard CTAB method. Subsequently, PCR was performed to amplify the genomic region encompassing the target sites using specific primers and 2 × Rapid Taq Master Mix (Vazyme Biotech Co., Ltd). The sequences obtained from Sanger sequencing were compared with the WT sequences to detect the presence of InDels. Additionally, the sequencing chromatograms were carefully examined to ensure there were no overlapping traces in the target regions surrounding the sgRNA, indicating homozygous mutations. Specific primers used in this process can be found in Supplementary Data Table S1.

### Analysis of conserved domain and protein structure

The conserved domains of genes were analyzed using CD-Search, available on the NCBI website (https://www.ncbi.nlm.nih.gov/Structure/cdd/wrpsb.cgi). CD-Search was employed by inputting the amino acid sequences of the respective genes to identify conserved domains. Furthermore, the protein structures of different genes were analyzed using SWISS-MODEL (https://swissmodel.expasy.org/).

### Determination of α-linolenic acid content

Flower bud clusters, comprising unopened flowers, were collected and subjected to freeze-drying using a freeze dryer (FD-C12N, Jingfu, Shanghai, China) until a constant weight was achieved. To measure α-LeA, one of the 11 fatty acid species, extraction was carried out followed by GC analysis using a GC-2014 gas chromatograph (Shimadzu, Kyoto, Japan). The extraction protocol and GC analysis conditions for α-LeA were performed as described in a previous study [51].

### Measurement of jasmonic acid and methyl jasmonate content

Jasmonates, including JA and MeJA, were extracted using an isopropanol–water–hydrochloric acid mixture, and their levels were quantified using HPLC–MS/MS. To ensure accuracy, the assay results were calibrated by incorporating an internal standard substance into the extract. In brief, flower bud clusters containing unopened flowers were detached from the plants and freeze-dried using a freeze dryer (FD-C12N, Jingfu, Shanghai, China) until a constant weight was achieved. The dried flower bud clusters were then ground into a powder and weighed, followed by transfer to 15-ml screw-cap tubes. To each tube, 10 ml of the isopropanol–water–hydrochloric acid mixed extract and 8 μl of a 1-μg/ml working solution of internal standards were added. The tubes containing the mixture were placed on a shaker at 4°C at a speed of 100 rpm for 30 min. Afterwards, 5 ml of dichloromethane was added, and the samples were further shaken for another 30 min at 4°C. Subsequently, the samples were centrifuged at 13 000 g for 5 min at 4°C. The solvent from the lower phase was carefully transferred to a screw-cap vial, concentrated using a nitrogen flow, and then redissolved in 0.4 ml of methanol. Finally, the samples were centrifuged at 13 000 g for 10 min at 4°C, and the resulting supernatant was passed through a 0.22-μm filter membrane for subsequent detection by HPLC–MS/MS.

### Paraffin sections

Fresh flower buds from both the WT and L1 were subjected to fixation using an ethanol–acetic acid fixative (3:1) for 2 days at room temperature. Following fixation, anthers were isolated and subjected to dehydration using a graded series of ethanol (75, 85, 90, 95, and 100%), followed by clearing with xylene. Subsequently, the anthers were infiltrated with paraffin wax and embedded in paraffin wax. Sections of ~5 μm thickness were obtained using a pathology slicer (RM2016, Shanghai Leica Instrument Co., Ltd). These sections were stained with toluidine blue and observed under a microscope (BX61, Olympus, Tokyo, Japan) equipped with a camera (DP71, Olympus, Tokyo, Japan) for capturing photographs.

### Measurement of pollen viability

Three blooming flowers were selected from both the WT and the DMs (L1 and L2), as well as the SMs (L3, L4). Petals and sepals were carefully removed, and the anthers were immersed in Alexander’s staining solution [52]. Following a 5-h staining period at room temperature, the staining results were examined using a microscope (BX61, Olympus, Tokyo, Japan) equipped with a camera (DP71, Olympus, Tokyo, Japan) for capturing photographs. Pollen grains displaying a dark purple color were considered active, while those appearing blue were deemed inactive. For each slide, five different fields of view were randomly selected and examined. In order to ensure statistical robustness, each field of view contained a minimum of 100 pollen grains.

### Application of methyl jasmonate

Flower bud clusters were subjected to treatment with 500 μM MeJA, either through immersion for 0.5 min per day or daily spraying. MeJA was dissolved in 0.05% aqueous Tween 20 solution. It is crucial to emphasize that opening flowers were not exposed to MeJA during the treatments.

### Construction of phylogenetic tree and sequence alignment

The amino acid sequence of the *AtDAD1* gene was subjected to sequence alignment by NCBI’s BLAST (https://blast.ncbi.nlm.nih.gov/Blast.cgi) to identify the homologous gene with the highest similarity to *AtDAD1* in species such as rice, maize, and soybean. The resulting data were utilized to construct a phylogenetic tree using MEGA (version 11.0.11, https://www.megasoftware.net/). To enhance the visual presentation of the phylogenetic tree, it was refined using iTOL (https://itol.embl.de/). Similarly, the sequence alignment result was enhanced using DNAMAN (version 9) for better clarity and interpretation.

### Statistical analysis

Statistical analysis was conducted using GraphPad Prism (version 9.0.0 for Windows, San Diego, CA, USA). One-way analysis of variance (ANOVA) followed by *post hoc* Student’s *t*-tests were employed to evaluate the significance of the experimental data.

## Supplementary Material

Web_Material_uhae139

## Data Availability

Data are available in the figures of the article and its supplementary materials.
