# Supplementary material for: A CRISPR/Cas9-induced male-sterile line facilitating easy hybrid production in polyploid rapeseed (Brassica napus)
Source: Hortic Res. 2024 May 28;11(7):uhae139. doi: 10.1093/hr/uhae139 (PMC11233878; doi:10.1093/hr/uhae139)
Supplement: Web_Material_uhae139 [file web_material_uhae139.docx]

**Supplementary information**

**A CRISPR/Cas9 Induced Male-Sterile Line Facilitating Easy Hybrid Production in Polyploid Rapeseed (*Brassica napus*)**

Mengxin Tu, Ruisen Wang, Wenhui Guo, Shiqi Xu, Yang Zhu, Jie Dong, Xiangtan Yao, and Lixi Jiang

**
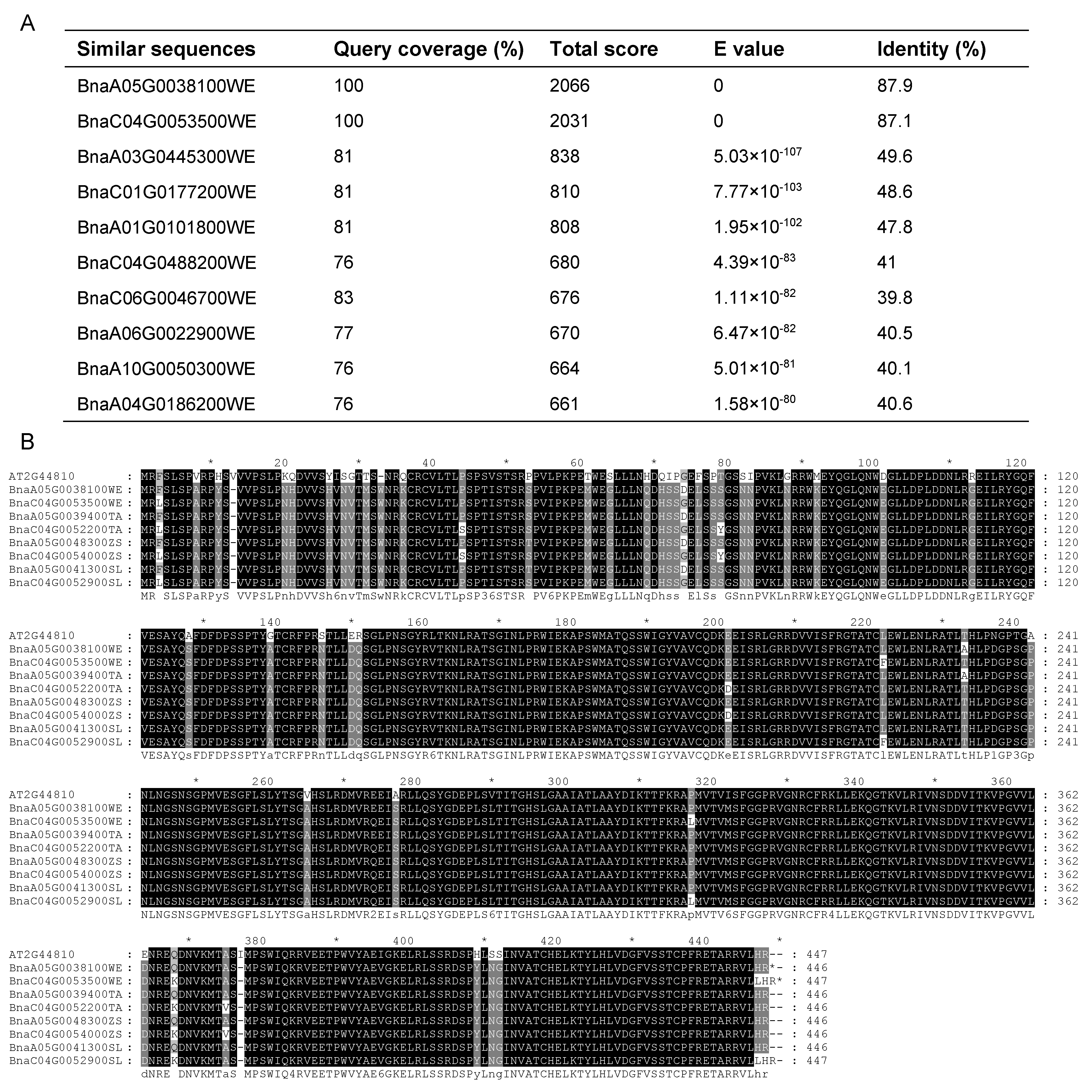
**

**Fig. S1 Identification of *DAD1* homologues in rapeseed and sequence conservation in different varieties.** (A) BLAST results of the *DAD1* gene in Westar with the amino acid sequence of *AtDAD1* as the reference. The BLAST was carried out in BnIR (<http://yanglab.hzau.edu.cn/BnIR/BLAST>). (B) Sequence alignment of DAD1 homologues in different varieties of Brassica napus and Arabidopsis thaliana. WE, Westar, a spring-ecotype rapeseed from Canada. TA, Tapidor, a winter-ecotype rapeseed from France. ZS, ZS11, a semi-winter-ecotype variety from China. SL, Shengli, a winter-ecotype rapeseed from China. Genome information is available in <https://yanglab.hzau.edu.cn/BnIR/download?module=genomics>.

**
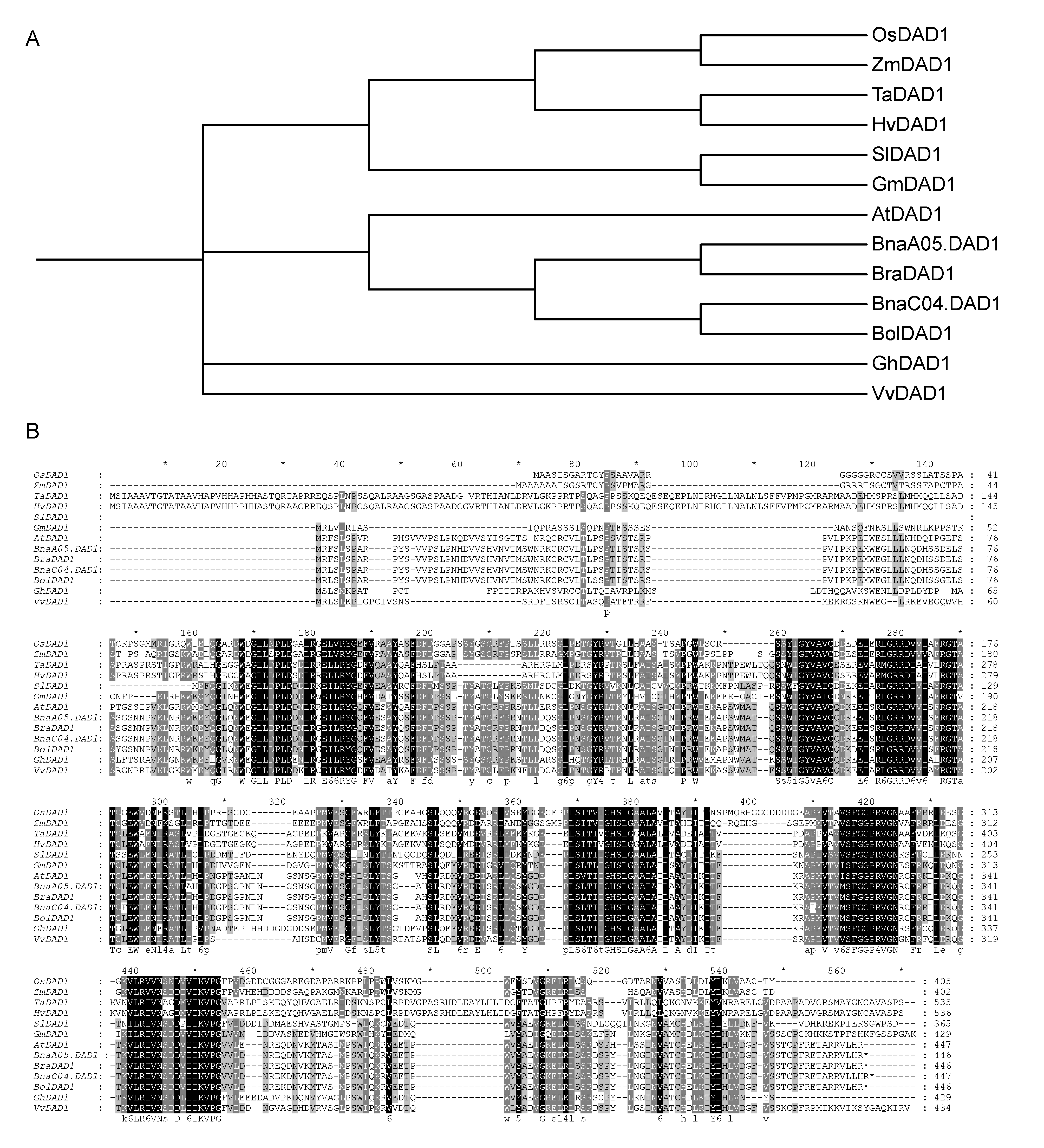
**

**Fig. S2** **Phylogenetic tree and sequence alignment of *DAD1* orthologue sequences identified in different species.** (A) Phylogenetic tree of *DAD1* orthologues are constructed using MEGA 11.0.11. Values at nodes represent the percentage of 1,000 bootstrap replicates. (B) Sequence alignment of *DAD1* orthologues. The amino acid sequence of *AtDAD1* was aligned in NCBI to identify *DAD1* orthologues. *DAD1* orthologues were identified in various plant species, including *Oryza sativa*, *Zea mays*, *Triticum aestivum*, *Hordeum vulgare*, *Solanum lycopersicum*, *Glycine max*, *Brassica rapa*, *Brassica napus*, *Brassica oleracea*, *Gossypium hirsutum* and *Vitis vinifera*.

**
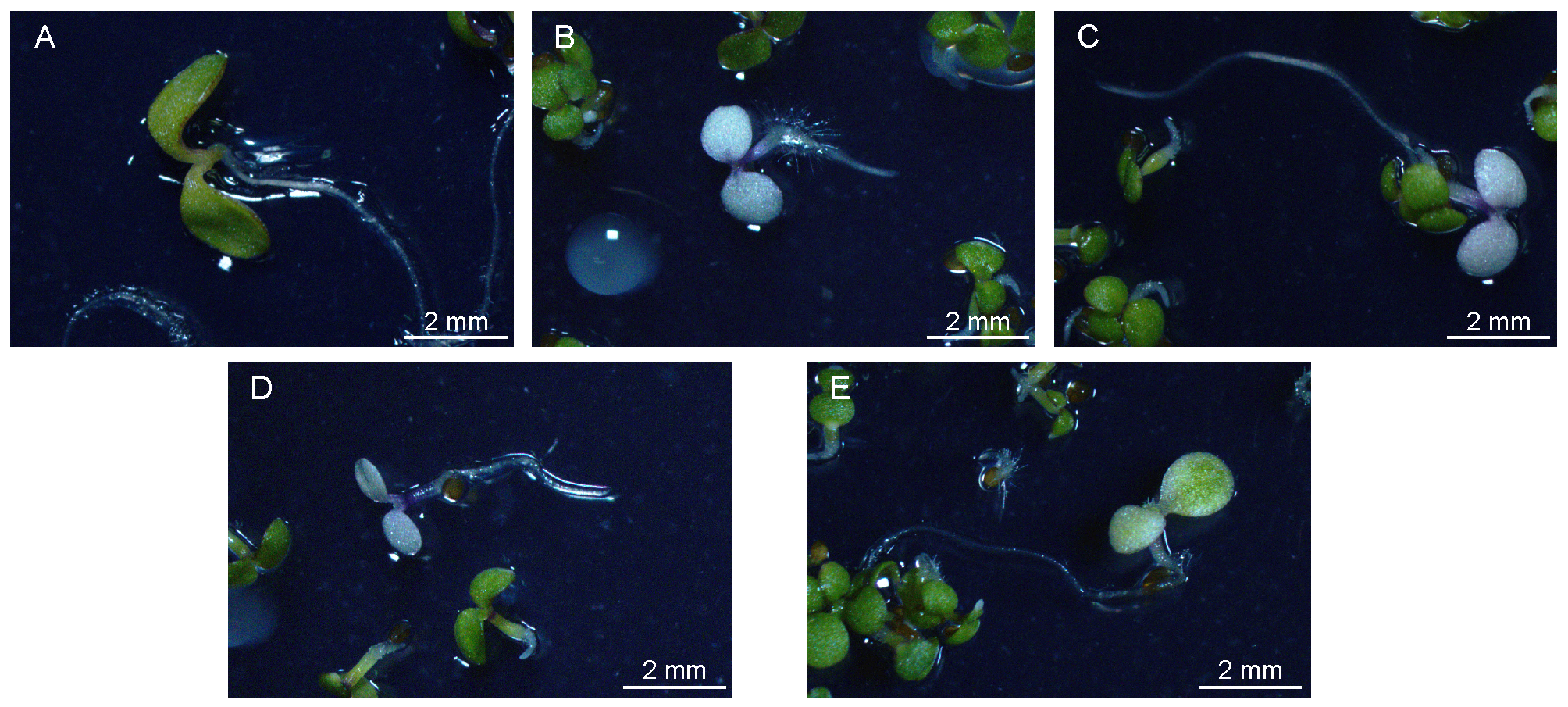
**

**Fig.** **S3** **Overexpression of *AtDAD1* and *BnDAD1* in *Arabidopsis*.**  (A) Wild type seedling exhibiting normal growth conditions 7 days after sowing. (B-D) T1 seedlings of overexpression line of 35S::*AtDAD1*(B), 35S::*BnaA05.DAD1*(C), and 35S::*BnaC04.DAD1*(D)all displayed a complete loss of green pigment 7 days after sowing, leading to their demise before true leaf formation. (E) T1 seedling of overexpression line of 35S::*BnaA05.DAD1* exhibited white patches in the leaves 7 days after sowing. Scale bar = 2mm.

**
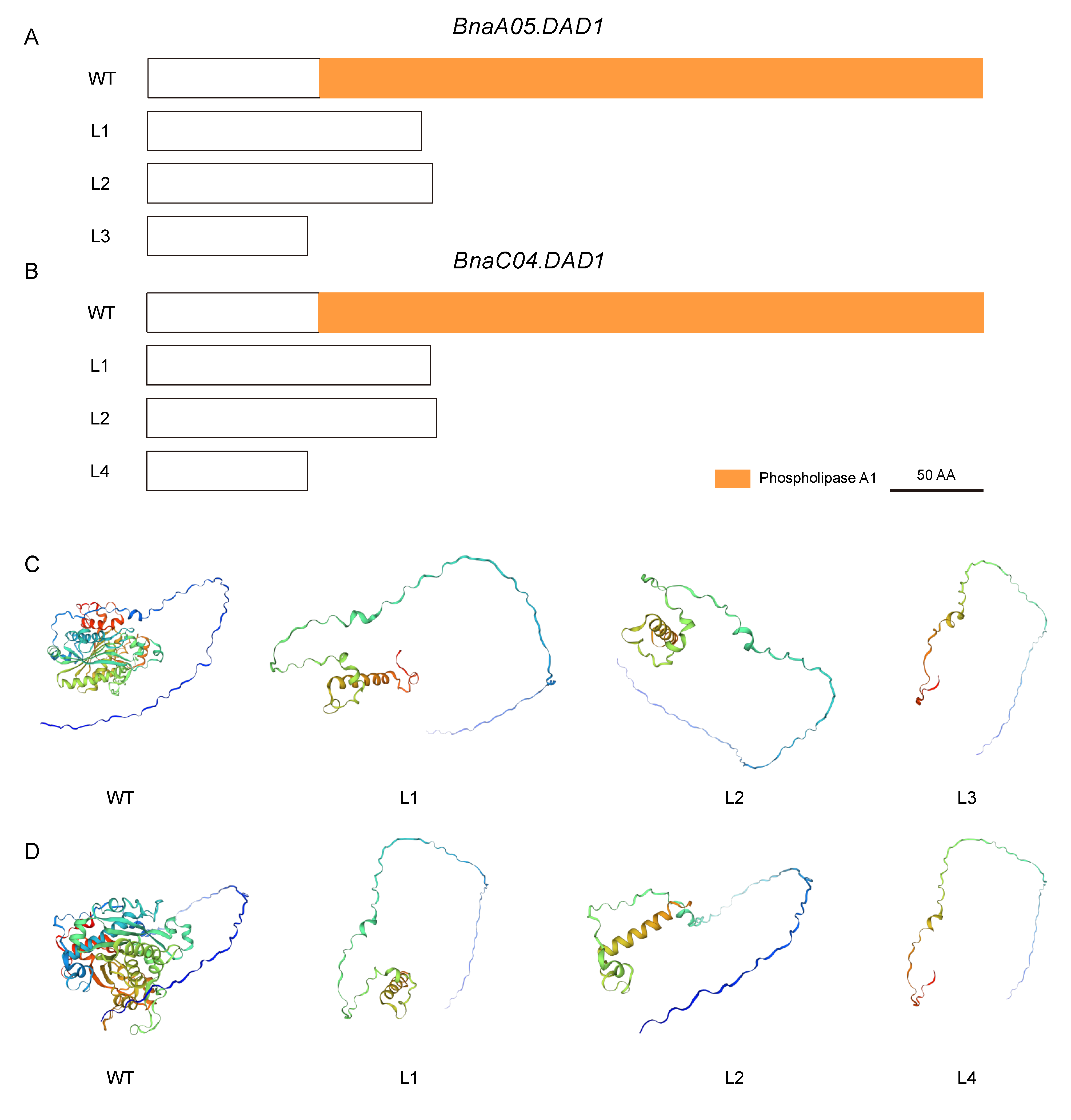
**

**Fig. S4** **Comparison of gene structure of *BnDAD1* between wild type (WT) and four mutants.** (A) Gene structure comparison of *BnaA05.DAD1* across WT, L1, L2, L3, and L4. (B) Gene structure comparison of *BnaC04.DAD1* across WT, L1, L2, L3, and L4. (C) Predicted protein structures of *BnaA05.DAD1* in WT, L1, L2, L3, and L4. (D) Predicted protein structures of *BnaC04.DAD1* in WT, L1, L2, L3, and L4.

**
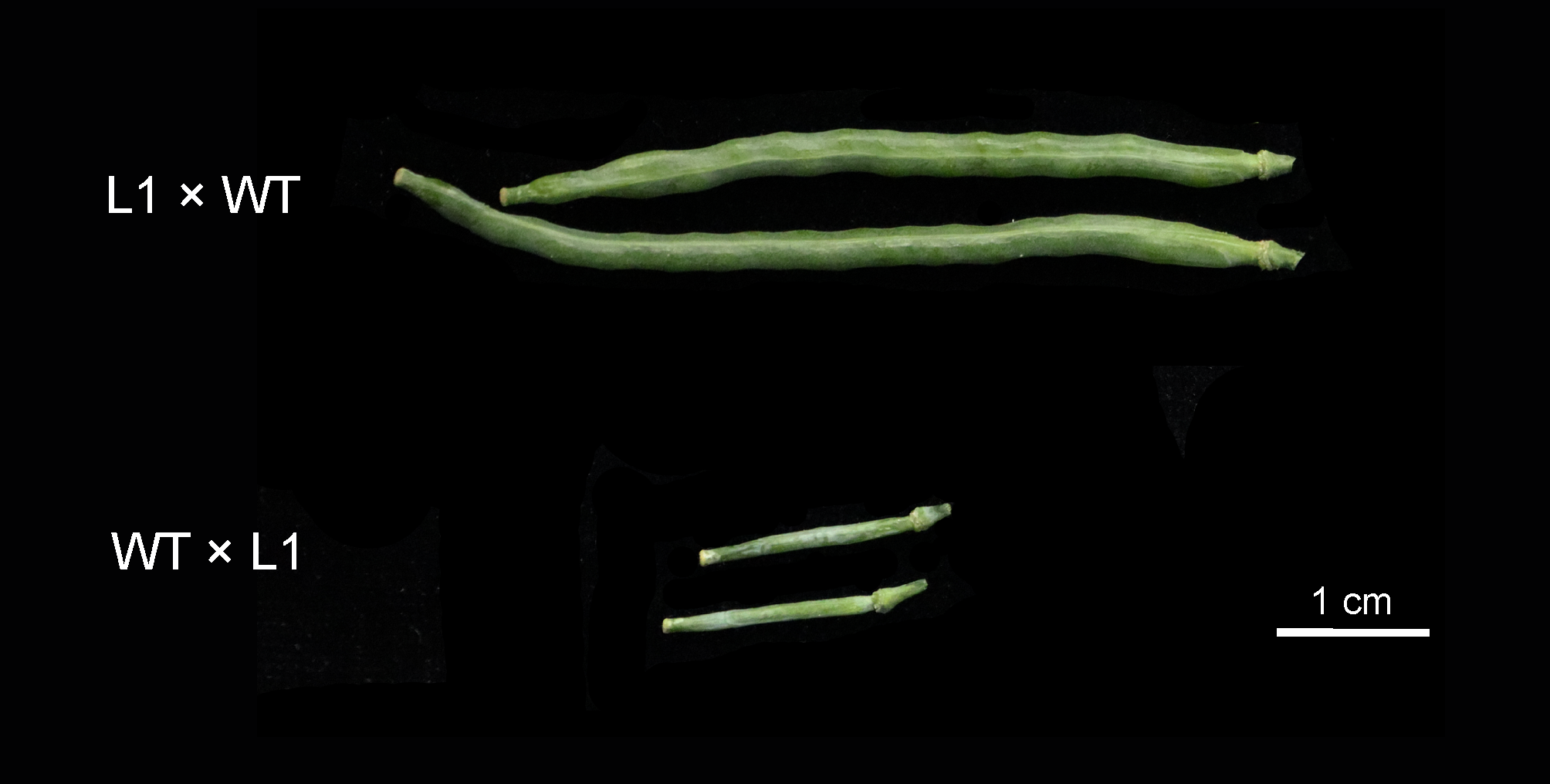
**

**Fig. S5 Siliques at 12 DAP of reciprocal pollinations between the L1 and the WT.** Scale bar = 1 cm.

**
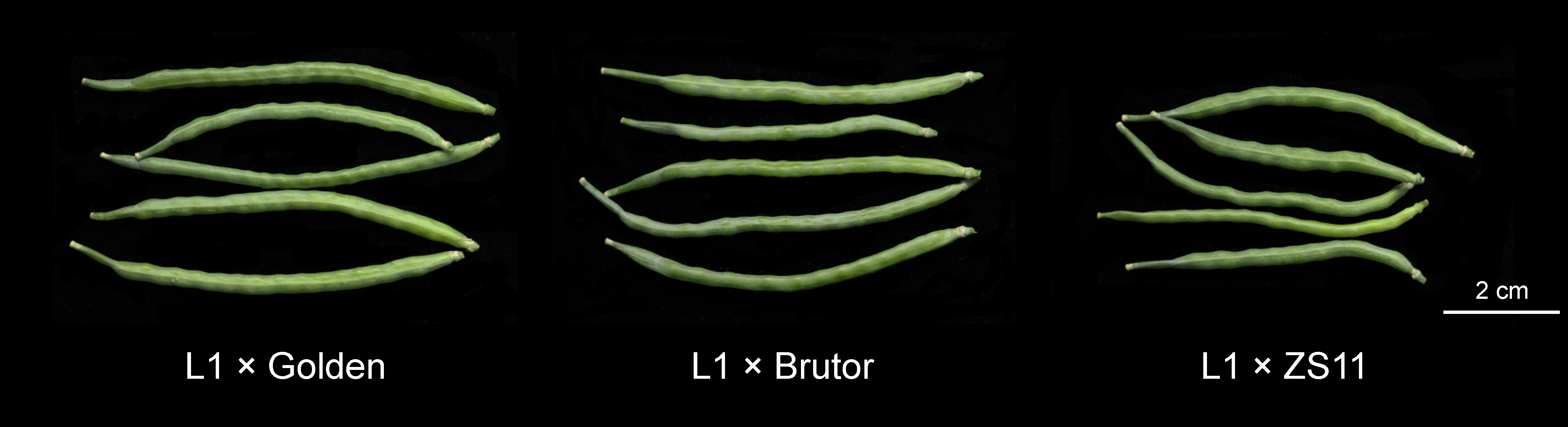
**

**Fig. S6 Siliques at 12 DAP of three hybrid combinations, L1 × Golden, L1 × Burtor, L1 × ZS11.** Golden and Brutor are spring-ecotype rapeseed, originated from Canada and France respectively. ZS11 is from China, and is a semi-winter ecotype. Scale bar = 2 cm.

**Table S1. Primers used in this study.**

| **Primer** | **Sequence (5' to 3')** | **Purpose** |
| --- | --- | --- |
| BnaDAD1-sgRNA01-f | GATTGTCCTCTGGTTCCAACAACC | CRISPR/Cas vectors construction |
| BnaDAD1-sgRNA01-r | AAACGGTTGTTGGAACCAGAGGAC |  |
| BnaDAD1-sgRNA02-f | GATTGTCCTCTGGTTCGAACAACC |  |
| BnaDAD1-sgRNA02-r | AAACGGTTGTTCGAACCAGAGGAC |  |
| BnaDAD1-sgRNA03-f | GATTGTACGGTCAGTTCGTGGAAT |  |
| BnaDAD1-sgRNA03-r | AAACATTCCACGAACTGACCGTAC |  |
| BnaDAD1-A5-f | ACCGTAAGTGTAGATGCGTA | Mutant detection primers |
| BnaDAD1-A5-r | TCCAGCTAGGCGCTTTCTCT |  |
| BnaDAD1-C4-f | ACCGTAAGTGTAGATGCGTG |  |
| BnaDAD1-C4-r | TCCAGCTAGGCGCTTTCTCA |  |
| qBnaDAD1-A5-f | AAACGGGTCTAACTCTGGGC | RT-qPCR primers |
| qBnaDAD1-A5-r | GCAGTCTCGAGATTTCTTCTCTC |  |
| qBnaDAD1-C4-f | CGAGACGTAGTCATCTCCTTCC |  |
| qBnaDAD1-C4-r | CCCAGAGTTAGACCCGTTTAGATTA |  |
| AtDAD1-ox-f | AGAGAACACCTGCAGGTCGACATGAGATTCTCTCTTTCTCC | Overexpression vectors construction |
| AtDAD1-ox-r | GGTACCCGGGGATCCTCTAGATCATCTATGGAGAACTCTCC |  |

To be continued

| **Primer** | **Sequence (5' to 3')** | **Purpose** |
| --- | --- | --- |
| BnaDAD1-A5-ox-f | AGAGAACACCTGCAGGTCGACATGAGATTCTCTCTTTCT |  |
| BnaDAD1-A5-ox-r | GGTACCCGGGGATCCTCTAGATCATCTATGGAGGACTCTTC |  |
| BnaDAD1-C4-ox-f | AGAGAACACCTGCAGGTCGACATGAGACTCTCTCTTTCT |  |
| BnaDAD1-C4-ox-r | GGTACCCGGGGATCCTCTAGATCATCTATGGAGGAGTACTC |  |
